# Mid-Term Outcome after Tricuspid Valve Replacement

**DOI:** 10.21470/1678-9741-2019-0215

**Published:** 2020

**Authors:** Yanmei Cheng, Shaoyan Mo, Keke Wang, Rui Fan, Yunqi Liu, Si Li, Xi Zhang, Shengli Yin, Yingqi Xu, Baiyun Tang, Zhongkai Wu

**Affiliations:** 1 Department of Cardiothoracic Surgery ICU, The First Affiliated Hospital of Sun Yat-sen University, Guangzhou, People’s Republic of China.; 2 Department of Cardiopulmonary Bypass, The First Affiliated Hospital of Sun Yat-sen University, Guangzhou, People’s Republic of China.; 3 Department of Cardiac Surgery, The First Affiliated Hospital of Sun Yat-sen University, Guangzhou, People’s Republic of China.; 4 Department of Echocardiography, The First Affiliated Hospital of Sun Yat-sen University, Guangzhou, People’s Republic of China.

**Keywords:** Cardiac Surgical Procedures, Tricuspid Valve, Risk factors, Intra-Aortic Balloon Pumping, Confidence Intervals, Survival Rate

## Abstract

**Objective:**

To evaluate the mid-term survival rate after tricuspid valve replacement (TVR).

**Methods:**

We retrospectively studied 110 consecutive patients who underwent TVR from January 2007 to November 2017. A survival analysis was performed with the Kaplan-Meier method and the log-rank test.

**Results:**

The median survival was 65.81 months. Mean age was 50 (range 39 to 59) years. Forty-eight patients (43.6%) were male, and 62 patients (56.4%) were female. Most of the patients (78.5%) were categorized into the New York Heart Association (NYHA) functional classes III/IV. Seventy-two patients (65.5%) had isolated TVR. Six-three patients (57.3%) had previously undergone heart surgery. The Kaplan-Meier survival rates at one year, three years, and five years were 59.0%±5%, 52.0%±6%, and 48.0%±6%, respectively. A Cox regression analysis demonstrated that the risk factors for mid-term mortality were advanced NYHA class (hazard ratio [HR] 2.430, 95% confidence interval [CI] 1.099-5.375, *P*=0.028), need for continuous renal replacement therapy (CRRT) treatment (HR 3.121, 95% CI 1.610-6.050, *P*=0.001), and need for intra-aortic balloon pump (IABP) treatment (HR 3.356, 95% CI 1.072-10.504, *P*=0.038).

**Conclusion:**

In TVR, impaired cardiac function before the operation and a need for CRRT or IABP treatment after the operation is independently associated with increased mid-term mortality.

**Table:** 

Abbreviations, acronyms & symbols				
**ASDR** **AVR** **AVS** **CI** **CRRT** **DVR** **eGFR** **EuroSCORE** **HR** **IABP** **MVR**	**= Atrial septal defect repair** **= Aortic valve replacement** **= Atrioventricular shunt** **= Confidence interval** **= Continuous renal replacement therapy** **= Double valve replacement** **= Estimated glomerular filtration rate** **= European System for Cardiac Operative Risk Evaluation** **= Hazard ratio** **= Intra-aortic balloon pump** **= Mitral valve replacement**		**NYHA** **PADR** **PBMV** **PBTV** **RV** **RVSP** **TAP** **TR** **TV** **TVR** **VSDR**	**= New York Heart Association** **= Patent ductus arteriosus repair** **= Percutaneous balloon mitral valvuloplasty** **= Percutaneous balloon tricuspid valvuloplasty** **= Right ventricular or right ventricle** **= Right ventricular systolic pressure** **= Tricuspid annuloplasty** **= Tricuspid regurgitation** **= Tricuspid valve** **= Tricuspid valve replacement** **= Ventricular septal defect repair**

## INTRODUCTION

Symptoms of tricuspid regurgitation (TR) are often nonspecific. However, surgical intervention for severe TR is only indicated in symptomatic patients^[1]^, and significant symptoms include signs of severe comorbidities. Therefore, patients referring for tricuspid valve (TV) surgery were often at a late stage, when right ventricular (RV) dysfunction has already occurred and TV repair has failed or is impossible, often risking a high mortality rate^[1]^.

In light of the high mortality rate in tricuspid valve replacement (TVR), an appropriate patient selection is crucially important for good clinical outcomes; however, the objective criteria are currently unavailable. Therefore, efforts have been made to determine predictors of clinical outcomes to perform a TVR procedure. The aim of our study was to identify the mid-term mortality risk factors for TVR, which might help in patient selection, yielding a satisfactory clinical outcome.

## METHODS

### Patient Selection

We retrospectively analyzed 110 patients who underwent TVR over a 10-year period, from January 2007 to November 2017. Ethics approval was obtained from the Medical Ethics Committee of the First Affiliated Hospital of Sun Yat-sen University. We included patients who underwent TVR either as an isolated procedure or in combination with other procedures. Late follow-up data were obtained from hospital records and from telephone contact with patients. The requirement for individual patient consent was waived because of the retrospective study design.

### Data Collection

Characteristics including sex, age, etiology, weight, height, diabetes mellitus, hypertension, coronary heart disease, atrial fibrillation, and New York Heart Association (NYHA) functional class were retrieved from hospital records. Laboratory parameters including hemoglobin, serum concentrations of bilirubin, alanine aminotransferase, creatinine, and blood urea nitrogen were collected. The estimated glomerular filtration rate (eGFR) was calculated using the Modification of Diet in Renal Disease equation: eGFR (mL/min/1.73 m^2^) = 175 × (serum creatinine)-1.234 × (age)-0.179 × (0.79, if female). Operative variables including prosthesis type, aortic cross-clamp and cardiopulmonary bypass times, and postoperative variables requirements for intra-aortic balloon pump (IABP) and continuous renal replacement therapy (CRRT) were collected. The European System for Cardiac Operative Risk Evaluation (EuroSCORE) II was calculated (http://www.euroscore.org/calc.html).

Two-dimensional transthoracic echocardiographic parameters were collected. Left ventricular ejection fraction was measured as guideline. Right atrial dimensions were estimated at end-diastole from an apical four-chamber view. Both right atrial length (referred to as the major dimension) and right atrial diameter (known as the minor dimension) at end-diastole were collected. The severity of TR was semiquantitatively graded as none, mild, moderate, moderately severe, severe, and extremely severe by quantifying TR velocity using color-flow Doppler from an apical view. The right ventricular systolic pressure (RVSP) was estimated based on continuous-wave Doppler measurements of TR jet velocity by using the modified Bernoulli equation^[2]^.

### Statistical Analysis

The categorical variables, which are presented as frequencies and percentages, were compared by using the χ^2^ test or Fisher’s exact test. The continuous variables, which are expressed as means ± standard deviation or medians with ranges, were compared by using Student’s unpaired *t*-test or Mann-Whitney U test. Analyses of survival were performed with the Kaplan-Meier method and the log-rank test. The overall mortality after triple valve operation was assessed by using the Cox proportional hazard model and was expressed as a hazard ratio (HR) with a 95% confidence interval (CI). Statistical significance was established with a *P*-value < 0.05.

## RESULTS

### Baseline Patient Characteristics

Table 1 summarizes the patients’ baseline demographic and clinical data. The mean patient age was 50 (range 39 to 59) years. Forty-eight patients (43.6%) were male, and 62 patients (56.4%) were female. The primary etiology for operative intervention was TV insufficiency caused by rheumatic heart disease in 41.8% (46/110) of the patients, followed by TV endocarditis in 21.8% (24/110) of the patients, degenerative valve disease in 20.9% (23/110) of the patients, and congenital heart disease in 15.5% (17/110) of the patients, with Ebstein’s anomaly in nine patients. The preoperative cardiac functions of the patients were classified as being NYHA functional class II (20.9%), III (71.8%), and IV (7.3%). Sixty-three (57.3%) patients had undergone previous cardiac operations. Chronic atrial fibrillation was present in 59 (53.6%) patients.

**Table 1:** Summary of the patients' baseline demographic profiles

Characteristics		All (n=110)
Age, years		50 (39, 59)
Male, n (%)		48 (43.6)
Heart rate, bpm		81 (75, 95)
Systolic blood pressure, mmHg		119 (110, 131)
Diastolic blood pressure, mmHg		72 (64, 80)
Body mass index, kg/m^2^		20.96 (19.23, 23.41)
Isolated TVR, n (%)		72 (65.5)
Etiology for intervention, n (%)	Infective endocarditis	24 (21.8)
	Rheumatic heart disease	46 (41.8)
	Degenerative valve disease	23 (20.9)
	Congenital heart disease	17 (15.5)
Previous heart disease, n (%)		63 (57.3)
NYHA, n (%)	II	23 (20.9)
	III	79 (71.8)
	IV	8 (7.3)
Coronary artery disease, n (%)		4 (3.6)
Hypertension, n (%)		11 (10)
Diabetes mellitus, n (%)		5 (4.5)
Atrial fibrillation, n (%)		59 (53.6)
Cardiopulmonary bypass time, min		130 (80, 182)
Aortic cross-clamping, n (%)		62 (56.4)
Prosthetic valve, n (%)		
Bioprosthesis		74 (67.3)
Mechanical prosthesis		36 (32.7)
**Laboratory examinations**		
Hemoglobin, g/L		124.5 (103,138)
Alanine aminotransferase, U/L		26.39±23.91
Bilirubin, umol/L		23.69±15.64
Blood urea nitrogen, mmol/L		6.54±4.82
Creatinine, µmol/L		77.25±32.55
eGFR, ml/min/1.73 m^2^		110.17±55.14
Right atrial diameter, mm		69.5 (58, 83)
Right atrial length, mm		50 (42, 68)
Left atrium, mm		47.5 (35, 54)
Left ventricular end-diastolic diameter, mm		47.5 (41, 52)
Left ventricular ejection fraction ≤ 55%		63.06±9.62
Right ventricular, mm		46.63±14.93
Mitral regurgitation ≥ moderate, n (%)		14 (12.7)
Mitral stenosis ≥ moderate, n (%)		15 (13.6)
Tricuspid regurgitation ≥ severe, n (%)		80 (72.7)
RVSP, mmHg		41 (32, 53)
CRRT, n (%)		27 (24.5)
IABP, n (%)		5 (4.5)
EuroSCORE II (%)		3.25 (2.42, 4.61)

CRRT=continuous renal replacement therapy; eGFR=estimated glomerular filtration rate; EuroSCORE=European System for Cardiac Operative Risk Evaluation; IABP=intra-aortic balloon pump; NYHA=New York Heart Association; RVSP=right ventricular systolic pressure; TVR=tricuspid valve replacement

Isolated TVR was performed in 72 (65.5%) patients. TVR concomitant procedures were performed in 28 (25.5%) patients. The mean cardiopulmonary bypass time was 130 minutes (range 80 to 182 minutes). Sixty-two (56.4%) procedures were performed with aortic cross-clamping. Bioprosthetic valves were implanted in 74 (67.3%) patients and mechanical valves in 36 (32.7%) patients.

### Echocardiographic Parameters

The baseline echocardiographic parameters were assessed by qualitative inspection of the 2D images. The left ventricular ejection fraction was 63.06%±9.62%. Left ventricular end-diastolic diameter was 47.5 mm (range 41 to 52 mm). RV diameter was 46.63±14.93 mm. Right atrial diameter was 69.5 mm (range 58 to 83 mm) and left atrial length was 50 mm (range 42 to 68 mm). The left atrial diameter was 47.5 mm (range 35 to 54 mm). The RVSP was 41 mmHg (range 32 to 53 mmHg). There were only 14 (12.5%) patients with equal to or more severe than moderate mitral regurgitation. The majority (72.7%, 80/110) of the patients had severe or more than severe TR.

Sixty-three (57.3%) patients had mitral valve replacement due to mitral valve disease, regurgitation, or stenosis. Eighteen (16.4%) patients had atrioventricular shunt. The detailed data of these two groups were presented in Table 2.

**Table 2:** Patients with atrioventricular shunt (AVS) and mitral valve disease.

	Patients with AVS (Total n=18) Indication for TVR			Patients with MVR procedure (Total n=66) Indication for TVR			
	Infective endocarditis (n=7)	Ebstein's anomaly (n=3)	Other (n=8)	Rheumatic heart disease (n=46)	Degenerative valve disease (n=15)	Congenital heart disease (n=3)	Infective endocarditis (n=2)
Previous heart procedure	n=2 (28.6%)	n=1 (33.3%)	n=6 (75.0%)	n=38 (82.6%)	n=10 (66.7%)	n=2 (66.7%)	n=1 (50%)
	1 VSDR+ASDR+ PADR with residual shunt	1 TAP+ASD	2 ASDR	1 AVR	5 MVR	1 ASDR	1 MVR+TAP
	1 VSDR		1 VSDR	13 DVR	5 DVR	1 VSDR +MVR	
			1 TAP+ASDR	1 PBMV; DVR			
			2 Fallot	19 MVR			
				1 MVR+TAP			
				1 MVR+ PBTV			
				2 PBMV			
Concomitant procedure	n=6 (85.7%)	n=2 (66.7%)	n=4 (50%)	n=16 (34.8%)	n=6 (40%)	n=3 (100%)	n=2 (100%)
	1 ASDR	1 ASDR	1 MVR	7 MVR	2 AVR	1 MVR	2 MVR
	1 Fallot	1 Fallot	2 VSDR	1 MVR+TAP	4 MVR	2 ASDR+MVR	
	1VSDR+ASDR+AVR		1 ASDR+MVR	4 AVR		3 VSDR	
	1 VSDR			4 DVR			
	1ASDR+VSDR+Gleen						
	1 AVR						
Mitral regurgitation	None	None	n=1 (12.5)	n=37 (17.5%)	n=11 (73.3%)	n=1 (33.3%)	n=2 (100%)
			1 Severe	31 Mild	7 Mild	1 Severe	
				3 Mediate	2 Mediate		
				3 Severe	2 Severe		
Mitral stenosis	None	None	None	n=12 (26.1%)	n=1	None	None
				1 Mild	1 Severe (stuck leaflet)		
				5 Mediate			
				6 Severe			
Right atrial length, mm	56.43±26.27	51.33±10.69	60.13±17.88	53.73±14.08	58.00±19.75	76.67±7.51	31.50±0.71
Right atrial diameter, mm	58.86±19.89	78.33±35.25	72.88±15.61	74.20±14.18	78.70±18.56	63.67±22.59	42.5±0.71
RVSP, mmHg	65.71±38.16	50.67±45.00	50.25±20.6	40.47±11.96	44.91±15.69	67.00±23.52	48.50±12.02
Death	2 (28.6%)	1 (33.3%)	4 (50.0%)	21 (45.7%)	3 (20.0%)	1 (33.3%)	1 (50.0%)

ASD=atrial septal defect repair; ASDR = Atrial septal defect repair; AVR=aortic valve replacement; DVR=double valve replacement; MVR=mitral valve replacement; PADR=patent ductus arteriosus repair; PBMV=percutaneous balloon mitral valvuloplasty; PBTV=percutaneous balloon tricuspid valvuloplasty; TAP=tricuspid annuloplasty; TVR=tricuspid valve replacement; RVSP=right ventricular systolic pressure; VSDR=ventricular septal defect repair

### Cox Regression Analysis

At the conclusion of our study, there was a median follow-up time of 65.81 months. The Cox univariate analysis revealed that bilirubin (*P*<0.001), blood urea nitrogen (*P*=0.031), creatinine (*P*=0.032), need for CRRT after surgery (*P*<0.001), need for IABP after surgery (*P*=0.006), advanced NYHA class (*P*<0.001), right atrial diameter (*P*=0.048), and cardiopulmonary bypass time (*P*=0.026) were risk factors for mid-term mortality (Table 3).

**Table 3:** Univariate and multivariate analysis of risk factors of mid-term mortality.

Variable		N	1-year survival (%)	3-year survival (%)	5-year survival (%)
Total		110	59.0±5	52.0±6	48.0 ±6
NYHA	II	23	82.6	71.2	71.2
	III	79	64.1	62.7	54.1
	IV	8	0	0	0
CRRT	No	83	75.5	72.7	62.9
	Yes	27	25.9	20.7	20.7
IABP	No	105	65.4	62.1	54.5
	Yes	5	20.0	20.0	20.0

CRRT=continuous renal replacement therapy; IABP=intra-aortic balloon pump; NYHA=New York Heart Association

In the adjusted Cox regression analysis, NYHA class (HR 2.430, 95% CI 1.099-5.375, *P*=0.028), need for CRRT treatment (HR 3.121, 95% CI 1.610-6.050, *P*=0.001), and need for IABP treatment (HR 3.356, 95% CI 1.072-10.504, *P*=0.038) remained to be independently associated with increased late mortality (Table 3).

### Survival at Follow-up

**Fig. 1:**
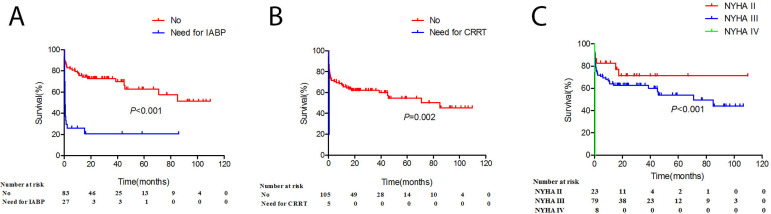
Survival curves A) according to the need for intra-aortic balloon pump (IABP) treatment, B) according to the need for continuous renal replacement therapy (CRRT) treatment, and C) according to New York Heart Association (NYHA) functional class.

Figure 1 shows the survival curves of the significant variables in both the univariate and multivariate analysis. Table 4 displays the time-related survival rates. The overall one-year, three-year, and five-year survival rates were 58%±5%, 51%±6%, and 47%±6%, respectively. The mortality rate was 100% for patients in NYHA class IV, whereas the one-year, three-year, and five-year survival rates were 62.3%, 61.0%, and 52.6%, respectively, in NYHA class III, and the one-year, three-year, and five-year survival rates were 83.3%, 72.5%, and 72.5%, respectively, in NYHA class II (*P*<0.001). Patients who needed CRRT treatment were more predisposed to death than patients who did not need CRRT treatment, with one-year, three-year, and five-year survival rates of 28%, 22.2%, and 22.4%, respectively, compared with one-year, thee-year, and five-year survival rates of 72.2%, 69.5%, and 55.3% respectively, in patients who did not need CRRT treatment (*P*=0.002). The one-year, three-year, and five-year survival rates for patients who needed IABP treatment after surgery were 20.0%, 20.0%, and 20.0% respectively, in contrast to 65.4%, 62.1%, and 54.5% in those patients who did not need IABP (*P*<0.001).

**Table 4:** Time-related survival rate for tricuspid valve replacement.

Characteristics	Univariate		Multivariate	
	HR (95% CI)	*P*-value	HR (95% CI)	*P*-value
Age, years	1.000 (0.980-1.020)	1.000		
Male, n	0.768 (0.435-1.358)	0.365		
Heart rate, bpm	1.010 (0.993-1.028)	0.258		
Systolic blood pressure, mmHg	0.983 (0.965-1.000)	0.051		
Diastolic blood pressure, mmHg	0.989 (0.965-1.014)	0.393		
Body mass index, kg/m^2^	0.970 (0.885-1.062)	0.511		
Isolated TVR, n	0.915 (0.502-1.669)	0.772		
Etiology for intervention, n	0.885 (0.658-1.189)	0.418		
Previous heart disease, n	0.821 (0.465-1.448)	0.495		
NYHA classification, n	4.547 (2.076-9.961)	<0.001	2.629 (1.151-6.001)	0.022
Coronary artery disease, n	1.396 (0.337-5.786)	0.646		
Hypertension, n	0.776 (0.279-2.164)	0.628		
Diabetes mellitus, n	0.796 (0.193-3.283)	0.752		
Atrial fibrillation, n	1.013 (0.574-1.788)	0.966		
Cardiopulmonary bypass time, min	1.004 (1.000-1.008)	0.026	1.003 (0.999-1.007)	0.194
Aortic cross-clamping, n	1.021 (0.575-1.814)	0.943		
Prosthetic valve, n	0.666 (0.349-1.269)	0.216		
Hemoglobin, g/L	0.992 (0.981-1.002)	0.122		
Alanine aminotransferase, U/L	1.005 (0.996-1.014)	0.263		
Bilirubin, umol/L	1.035 (1.020-1.049)	<0.001	1.017 (0.999-1.035)	0.060
Blood urea nitrogen, mmol/L	1.039 (1.003-1.076)	0.031	1.041 (0.987-1.097)	0.138
Creatinine, µmol/L	1.007 (1.001-1.013)	0.032	1.004 (0.995-1.013)	0.360
eGFR, ml/min/1.73 m^2^	0.996 (0.989-1.003)	0.238		
Right atrial diameter, mm	1.016 (1.000-1.032)	0.048	1.004 (0.985-1.022)	0.686
Right atrial length, mm	1.010 (0.995-1.025)	0.206	1.010 (0.986-1.034)	0.421
Left atrium, mm	0.986 (0.967-1.006)	0.183		
Left ventricular ejection fraction ≤ 55%	0.566 (0.263-1.219)	0.146		
Left ventricular end-diastolic diameter, mm	1.001 (0.968-1.034)	0.965		
Right ventricle, mm	1.028 (1.002-1.056)	0.037	0.983 (0.941-1.026)	0.434
Mitral regurgitation ≥ moderate, n	1.166 (0.523-2.601)	0.707		
Mitral stenosis ≥ moderate, n	1.180 (0.529-2.633)	0.686		
Tricuspid regurgitation ≥ severe, n	0.896 (0.480-1.671)	0.729		
RVSP, mmHg	0.996 (0.980-1.012)	0.632		
CRRT, n	4.370 (2.425-7.874)	<0.001	3.198 (1.653-6.187)	0.001
IABP, n	4.558 (1.550-13.401)	0.006	3.895 (1.168-12.985)	0.027
EuroSCORE II (%)	1.036 (0.921-1.166)	0.556		

CI=confidence interval; CRRT=continuous renal replacement therapy; eGFR=estimated glomerular filtration rate; EuroSCORE=European System for Cardiac Operative Risk Evaluation; HR=hazard ratio; IABP=intra-aortic balloon pump; NYHA=New York Heart Association; RVSP=right ventricular systolic pressure; TVR=tricuspid valve replacement

## DISCUSSION

Advanced NYHA functional classes in admission and the need for CRRT or IABP treatment were independent risk factors strongly associated with increased mid-term mortality after TVR. The overall one-year, three-year, and five-year survival rates were 58%±5%, 51%±6%, and 47%±6%, respectively. The results were similar to other published studies with reported five-year actuarial survival rates of 41.6% to 74%^[3-11]^.

The pathology of TR was attributed to damage of TV or to tricuspid annular dilation and ventricular enlargement, leading to improper TV leaflet coaptation^[12]^. Primary regurgitation results from lesions of the TV apparatus itself, such as endocarditis, rheumatic heart disease, or congenital malformation. More often, TR is functional and secondary to mitral regurgitation or stenosis, leading to increased left atrial pressure, secondary pulmonary hypertension, and finally functional TR. It was recommended that severe TR should be corrected at the time of the initial mitral valve surgery, with a class I recommendation suggested by two guidelines^[13,14]^. The American College of Cardiology guideline gives a class IIa recommendation for TV annuloplasty in patients with tricuspid annular diameter > 40 mm or 21 mm/m^2^ diameter indexed to body surface area measured by 2D echocardiography or > 70 mm diameter measured by direct TV inspection during mitral valve surgery, even in the absence of functional TR (class Ⅱa)^[14]^.

Organic TV disease often requires TVR surgery. Functional TR can usually be corrected with TV repair^[15]^. Nonring suture annuloplasty bicuspidalization (*i.e*., plication of the posterior leaflet) is often performed in rheumatic heart disease patients, whereas De Vega annuloplasty (*i.e*., plication of the annulus surrounding the anterior and posterior leaflets) is usually performed in patients with severe tricuspid annular dilation, remodeling the annulus by maintaining TV a more physiologic annulus^[15]^.

In correction of functional TR, ring annuloplasty yields a better outcome compared to nonring repair^[15,16]^. Functional TR was previously thought to diminish after left-sided valve surgery^[17]^. Moderate-to-severe TR was an independent risk factor for adverse event and worse survival after mitral valve replacement^[18]^. In cases of less severe TR, left uncorrected at mitral valve surgery, 25% of the patients might worsen toward to severe TR across time and had worse outcome and survival^[14]^. So, aggressive prophylactic TV repair was suggested to be performed in patients undergoing mitral valve replacement regardless of TR severity. However, McCarthy et al.^[16]^ documented early failure in all types of TV annuloplasty repair in 14% of 790 annuloplasty patients within one month, furthermore, more severe preoperative TR was significantly associated with higher late TR risk. Nakanishi et al. revealed residual TR significantly associated with worse survival^[19]^. All efforts should be made to eliminate this residual functional TR after left-sided heart operations^[20]^.

Late TR after mitral valve replacement is often isolated and occurs in the absence of significant left heart disease^[1]^. However, in TR, isolated TV surgery is only recommended in symptomatic patients or patients who had progressed RV dilatation^[21]^. As a result, isolated TVR to correct TR is a difficult problem due to the late referral and manifestations of damaged RV function. RV failure is an outcome determinant in TV surgery. Seventy-two (65%) patients in our study underwent isolated TVR surgery. Until now, data comparing tricuspid annuloplasty with TVR in functional TR after left-sided heart valve surgery has been scarce. Mangoni et al.^[22]^ supported the idea of performing TVR rather than repair because of the high risk of recurrence of significant TR after repair.

Evidence have accumulated that symptoms of RV failure, such as hepatomegaly and icterus^[4]^, anasarca^[9]^, ascites, and high preoperative bilirubin level^[23]^, were associated with an increased mortality risk after TVR surgery. In patients who underwent cardiac surgery, the Model for End-stage Liver Disease and EuroSCORE were demonstrated to be useful in mortality prediction^[24]^. In 40 isolated TVR patients, a marginal association was demonstrated between logistic EuroSCORE I and mortality risk (HR 1.06, *P*=0.001)^[25]^ We calculated the EuroSCORE II, it takes liver function into account and its calculation used clearance instead of serum creatinine, resulting in a more accurate measure of renal function compared to EuroSCORE I, however, no association was found with mortality in univariate or multivariate analysis. In Cox univariate analysis, we found out that higher preoperative bilirubin, blood urea nitrogen, and creatinine levels were significant mortality risk factors. However, this significance disappeared after adjustment of other risk factors.

It is recognized that not the surgery itself that is difficult, but rather the RV dysfunction after the restoration of competence to the insufficient TV that matters^[1]^. The restoration of valve competence via the correction of TR may lead to RV decompensation when RV cannot sustain the pressure and/or volume overload after the correction^[26]^.

Our current study demonstrated that patients ranked as advanced NYHA classes had significantly higher mid-term mortality risk, a trend also observed in other published studies^[6,27-29]^. In our study, 87 (79.1%) patients were in NYHA functional classes III/IV. The eight NYHA IV patients died immediately after operation. Patient selection is crucial for better surgical outcomes. Those NYHA IV patients should be modulated by optimizing medical support. No signs of cardiac functional improvement prior to surgery might serve as a contraindication of TVR. Recently, Hamandi et al.^[30]^ emphasized the importance of the periprocedural management of RV failure in TV surgery by proposing that the improvement of RV function can provide better outcomes. This can provide important guidance in the management of TV surgery patients.

Long-term TR leads to further RV dilation, TV annular dilation, and finally causes RV dysfunction^[31,32]^. Sharma et al. reported a 40% rate of worsening renal function in RV failure patients^[33]^. Patients with RV failure were also reported to be more predisposed to CRRT compared with non-RV failure patients^[34]^. Their further research revealed that renal deterioration was significantly associated with RV failure^[35]^. Furthermore, it was demonstrated to be a predictor of long-term mortality and morbidity outcomes in RV failure patients^[36]^. Five patients need IABP mechanical support as a result of low cardiac output and unstable hemodynamics. Two patients had moderate mitral stenosis before TVR and three patients had left ventricular ejection fraction less than 55% before surgery. Four patients who required IABP support died.

In the absence of gradient between the pulmonary valve and the RV outflow tract, the RVSP was assumed to be equivalent to the pulmonary artery systolic pressure^[2]^. In functional TR, elevated pulmonary artery systolic pressure is a major cause of TR, furthermore, TR progresses and regresses with the fluctuation of PASP^[37]^. There were studies correlating elevated pulmonary artery systolic pressure with increased early and long-term mortalities after TVR^[6,23]^. However, Mutlak et al.^[38]^ found no correlation of pulmonary artery systolic pressure with TR severity. In our study, we did not find any association between RVSP and mortality. Endocarditis has a high mortality in the immediate postoperative course. In endocarditis patients, five-year survival after TVR is 36.8%. Four (16.7%) of the 24 infective endocarditis patients were intravenous drug abusers, and they all died of infection. Most of endocarditis patients died of blood-borne disseminated lung abscess or other severe infections.

Until now, there was no clear superiority of one prosthesis over another, the decision should be individualized to the patient. The optimal choice of valve type in TVR is still controversial. Most studies have not demonstrated one valve type to outperform another in both early and late survival rates^[4,8,11]^. Our study also demonstrated that prosthesis type has no influence on survival. For the prosthesis choice, it was more often based on the surgeon’s discretion and preference. Clinicians need to take time-related adverse events into consideration. In our institute, warfarin was initiated on postoperative day one or two in all stable non-bleeding patients, and warfarin dose was adjusted to maintain a target International Normalized Ratio of 1.8 to 2.5. Postoperative anticoagulation therapy in bioprosthetic replacement terminated after six months, whereas mechanical prostheses replacement required lifelong anticoagulation therapy. Bioprostheses have usually been advocated due to a lower demand of anticoagulation^[4,39]^. However, the degeneration of the bioprostheses and the higher reoperation rates are barriers for the application of this prosthesis type^[2,4,40-42]^; hence, the choice of a mechanical prosthesis has been preferred. There has also been a study demonstrating that no difference in reoperation rates was found between the prosthetic valves^[8]^.

However, with advanced surgical and transcatheter therapies, the transcatheter therapies with decreased risk of adverse events are applied to TV. As the management of valvular heart disease progresses, transcatheter edge-to-edge repair is reported to be performed in selected inoperable patients with severe TR^[43]^. Though difficult to anchor a transcatheter valve for TV, the Heterotopic Implantation of the Edwards-Sapien XT Transcatheter Valve in the Inferior VEna Cava for the Treatment of Severe Tricuspid Regurgitation - HOVER trial set out to explore the efficacy of caval valve implant in severe TR^[44]^, providing promising future for TR treatment.

Our current study supports the same idea, that poor cardiac function was the predominant cause of poor outcomes of TVR.

### Limitations

First, this study was subject to the limitations that are inherent to a retrospective analysis of observational data. RV function was not systematically evaluated at either preoperative or postoperative time points. The quantitative echocardiography parameters reflecting RV systolic function, such as tricuspid annular plane systolic excursion and inferior vena cava size, were not estimated. Moreover, more precise estimates of RV function, such as 3D echocardiography or magnetic resonance imaging with excellent ability to quantitate RV volumes and ejection fraction, may help to identify those patients who will not benefit from TVR. Finally, this is a single center study with small sample size. Further cohort studies with large sample size are needed.

## CONCLUSION

In TVR, advanced NYHA classes before operation and need for CRRT or IABP treatment after operation are mortality risk factors for mid-term outcomes.

**Table:** 

Authors' roles & responsibilities	
YC SM KW RF YL SL XZ SY YX BT ZW	Substantial contributions to the conception or design of the work; or the acquisition, analysis, or interpretation of data for the work; drafting the work or revising it critically for important intellectual content; final approval of the version to be published The acquisition of data for the work; final approval of the version to be published The acquisition of data for the work; final approval of the version to be published The acquisition of data for the work; final approval of the version to be published Analysis, or interpretation of data for the work; final approval of the version to be published The acquisition of data for the work; final approval of the version to be published Interpretation of data for the work; final approval of the version to be published Interpretation of data for the work; final approval of the version to be published Interpretation of data for the work; final approval of the version to be published Substantial contributions to the conception or design of the work; revised it critically; final approval of the version to be published Substantial contributions to the conception or design of the work; revised it critically; final approval of the version to be published
